# Supplementary material for: Single Nucleotide Polymorphism Induces Divergent Dynamic Patterns in CYP3A5: A Microsecond Scale Biomolecular Simulation of Variants Identified in Sub-Saharan African Populations
Source: Int J Mol Sci. 2021 Jul 21;22(15):7786. doi: 10.3390/ijms22157786 (PMC8346100; doi:10.3390/ijms22157786)
Supplement: Supplementary file 1 [file ijms-22-07786-s001.zip › ijms-1243567-supplementary.pdf]

Single nucleotide polymorphism induces divergent dynamic patterns in CYP3A5: a microsecond scale biomolecular simulation of variants identified in Sub-Saharan African populations (Supplementary materials)

Houcemeddine Othman[1], Jorge da Rocha[1,2], and Scott Hazelhurst[1,3]

[1]Sydney Brenner Institute for Molecular Bioscience, Faculty of Health Sciences, University of the Witwatersrand, Johannesburg, South Africa.
[2]Division of Human Genetics, National Health Laboratory Service, and School of Pathology, Faculty of Health Sciences, University of the Witwatersrand, Johannesburg, South Africa.
[3]School of Electrical and Information Engineering, University of the Witwatersrand, Johannesburg, South Africa.

Supplementary material 1: CYP3A5 drugs

This list of drugs was compiled from DrugBank database.

Drug	type
Alfentanil	substrate
Alprazolam	substrate
Ambrisentan	substrate
Amitriptyline	substrate
Amprenavir	substrate
Anastrozole	substrate
Apixaban	substrate
Apomorphine	substrate
Argatroban	substrate
Aripiprazole	substrate
Aripiprazole lauroxil	substrate
Artemether	substrate
Astemizole	substrate
Atorvastatin	substrate
Axitinib	substrate
Azelastine	substrate
Azimilide	substrate
Benidipine	substrate
Bimatoprost	substrate
Buprenorphine	substrate
Buspirone	substrate
Cabazitaxel	substrate

Cerivastatin	substrate
Cethromycin	substrate
Chloroquine	substrate
Chlorpheniramine	substrate
Cilostazol	substrate
Cisapride	substrate
Clindamycin	substrate
Clonidine	substrate
Clopidogrel	substrate
Copanlisib	substrate
Cyclophosphamide	substrate
Cyclosporine	substrate
Daclatasvir	substrate
Dapsone	substrate
Dasatinib	substrate
Deutetrabenazine	substrate
Dexloxiglumide	substrate
Diazepam	substrate
Disulfiram	substrate
Docetaxel	substrate
Domperidone	substrate
Doravirine	substrate
Dutasteride	substrate
Elexacaftor	substrate
Eplerenone	substrate
Erlotinib	substrate
Estradiol	substrate
Estradiol acetate	substrate
Estradiol benzoate	substrate
Estradiol cypionate	substrate
Estradiol dienanthate	substrate
Estradiol valerate	substrate
Estrone	substrate
Ethinylestradiol	substrate
Ethosuximide	substrate
Etoposide	substrate
Felodipine	substrate
Fenfluramine	substrate
Finasteride	substrate
Fluoxetine	substrate
Flutamide	substrate
Fluvastatin	substrate
Gefitinib	substrate
Glyburide	substrate
Halofantrine	substrate
Haloperidol	substrate
Hydrocortisone	substrate
Hydrocortisone aceponate	substrate
Hydrocortisone acetate	substrate
Hydrocortisone butyrate	substrate
Hydrocortisone cypionate	substrate

Hydrocortisone phosphate	substrate
Hydrocortisone probutate	substrate
Hydrocortisone valerate	substrate
Hydroxyprogesterone caproate	substrate
Hydroxyzine	substrate
Ibrutinib	substrate
Ifosfamide	substrate
Iloperidone	substrate
Irinotecan	substrate
Istradefylline	substrate
Ivacaftor	substrate
Lapatinib	substrate
Lemborexant	substrate
Lercanidipine	substrate
Levonorgestrel	substrate
Lidocaine	substrate
Loratadine	substrate
Lorlatinib	substrate
Loxoprofen	substrate
Metronidazole	substrate
Mifepristone	substrate
Mycophenolate mofetil	substrate
Naldemedine	substrate
Nateglinide	substrate
Nevirapine	substrate
Nifedipine	substrate
Nisoldipine	substrate
Nitrendipine	substrate
Nortriptyline	substrate
Olaparib	substrate
Omega-3-carboxylic acids	substrate
Ondansetron	substrate
Oxybutynin	substrate
Oxycodone	substrate
Paclitaxel	substrate
Paliperidone	substrate
Paritaprevir	substrate
Pentamidine	substrate
Perampanel	substrate
Pimozide	substrate
Ponatinib	substrate
Praziquantel	substrate
Progesterone	substrate
Propranolol	substrate
Quetiapine	substrate
Quinacrine	substrate
Relugolix	substrate
Risdiplam	substrate
Rivaroxaban	substrate
Romidepsin	substrate
Salmeterol	substrate

Saxagliptin	substrate
Selpercatinib	substrate
Selumetinib	substrate
Sildenafil	substrate
Simvastatin	substrate
Sirolimus	substrate
Sorafenib	substrate
Sunitinib	substrate
Tacrolimus	substrate
Tadalafil	substrate
Tamoxifen	substrate
Tazemetostat	substrate
Tegafur	substrate
Tenapanor	substrate
Teniposide	substrate
Terfenadine	substrate
Testosterone	substrate
Testosterone cypionate	substrate
Testosterone enanthate	substrate
Testosterone undecanoate	substrate
Tezacaftor	substrate
Trastuzumab emtansine	substrate
Trazodone	substrate
Tretinoin	substrate
Triazolam	substrate
Udenafil	substrate
Umifenovir	substrate
Valbenazine	substrate
Valproic acid	substrate
Vardenafil	substrate
Vicriviroc	substrate
Vincristine	substrate
Vortioxetine	substrate
Zaleplon	substrate
Zanubrutinib	substrate
Zonisamide	substrate
Midazolam	substraes
Ambroxol acefyllinate	not specified
Brigatinib	not specified
Foretinib	not specified
Dolutegravir	not specified
Elagolix	not specified
Mirodenafil	not specified
Moxisylyte	not specified
Myrrh	not specified
Rhein	not specified
Acalabrutinib	inhibitor
Amlodipine	inhibitor
Boceprevir	inhibitor
Cannabidiol	inhibitor
Cenobamate	inhibitor

Chloramphenicol	inhibitor
Cimetidine	inhibitor
Ciprofloxacin	inhibitor
Clarithromycin	inhibitor
Clofazimine	inhibitor
Cobicistat	inhibitor
Crizotinib	inhibitor
Curcumin sulfate	inhibitor
Delavirdine	inhibitor
Diltiazem	inhibitor
Ditiocarb	inhibitor
Dronedarone	inhibitor
Elbasvir	inhibitor
Erythromycin	inhibitor
Fluconazole	inhibitor
Fluticasone propionate	inhibitor
Fluvoxamine	inhibitor
Gestodene	inhibitor
Glecaprevir	inhibitor
Idelalisib	inhibitor
Imatinib	inhibitor
Indinavir	inhibitor
Isavuconazole	inhibitor
Itraconazole	inhibitor
Ketoconazole	inhibitor
Letermovir	inhibitor
Lonafarnib	inhibitor
Lopinavir	inhibitor
Mebendazole	inhibitor
Medical Cannabis	inhibitor
Methylene blue	inhibitor
Mibefradil	inhibitor
Nabiximols	inhibitor
Nefazodone	inhibitor
Nelfinavir	inhibitor
Nicardipine	inhibitor
Olanzapine	inhibitor
Osilodrostat	inhibitor
Oxymetholone	inhibitor
Phenelzine	inhibitor
Pibrentasvir	inhibitor
Remoxipride	inhibitor
Ritonavir	inhibitor
Rucaparib	inhibitor
Safinamide	inhibitor
Saquinavir	inhibitor
Seproxetine	inhibitor
Temsirolimus	inhibitor
Troglitazone	inhibitor
Troleandomycin	inhibitor
Tucatinib	inhibitor

Verapamil	inhibitor
Voriconazole	inhibitor
Amcinonide	Inducer
Armodafinil	Inducer
Asunaprevir	Inducer
Beclomethasone dipropionate	inducer
Betamethasone	inducer
Betamethasone phosphate	inducer
Budesonide	inducer
Carbamazepine	inducer
Clobetasol	inducer
Clobetasol propionate	inducer
Clocortolone acetate	inducer
Cloprednol	inducer
Cortisone	inducer
Cortisone acetate	inducer
Cortivazol	inducer
Daunorubicin	inducer
Deflazacort	inducer
Dexamethasone	inducer
Dexamethasone acetate	inducer
Dexamethasone isonicotinate	inducer
Difluocortolone	inducer
Efavirenz	inducer
Enzalutamide	inducer
Fluocinonide	inducer
Fluocortolone	inducer
Fluorometholone	inducer
Fluprednidene	inducer
Fluprednidene acetate	inducer
Fluticasone	inducer
Fluticasone furoate	inducer
Halometasone	inducer
Icotinib	inducer
Medrysone	inducer
Melengestrol acetate	inducer
Meprednisone	inducer
Methylprednisolone	inducer
Methylprednisolone hemisuccinate	inducer
Midostaurin	inducer
Modafinil	inducer
Mometasone	inducer
Mometasone furoate	inducer
Phenobarbital	inducer
Phenytoin	inducer
Pralsetinib	inducer
Prednisolone hemisuccinate	inducer
Prednisolone phosphate	inducer
Prednisone	inducer
Prednisone acetate	inducer
Prednylidene	inducer

Quinine	inducer
Reserpine	inducer
Rifampicin	inducer
Rifapentine	inducer
Rimexolone	inducer
Thalidomide	inducer
Thiopental	inducer
Triamcinolone	inducer
Oxcarbazepine	inducer
Paramethasone	inducer

Supplementary material 2: Mapping CYP3A5 amino acid variants on the protein structure

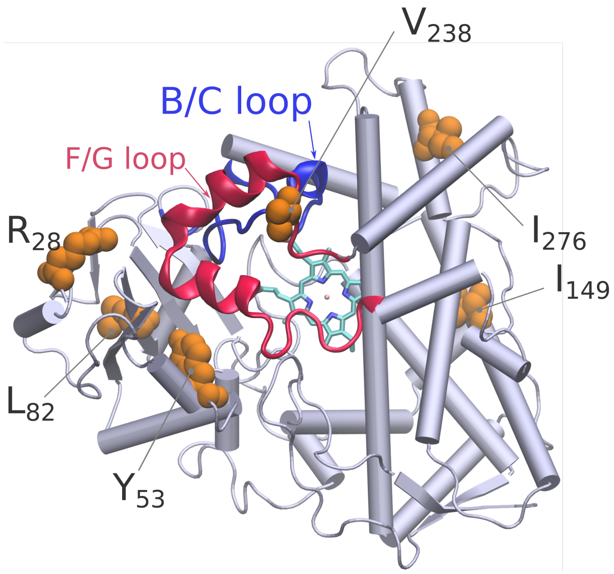

Figure S1: Position of the amino acids corresponding to the missense variants on the 3D structure of CYP3A5. The figure was generated using the Visual Molecular Dynamics program (VMD).

Supplementary material 3: Assessing the convergence of the molecular dynamics trajectory

To assess the convergence of each trajectory we have made a clustering analysis over all the snapshots using cpptraj. We choose to make a hierarchical clustering using a cutoff value of 2 Å. The cumulative number of clusters as a function of time was calculated as well as the evolution of informational entropy according to the following formula.

$$H = -\sum_{i=1}^{n} p_i log(p_i)$$

where p_i is the probability of the ith found cluster, as a function of simulation time.

The wild type form, Y53C, I149T, and I276T had shown a constant number of clusters of 1 since

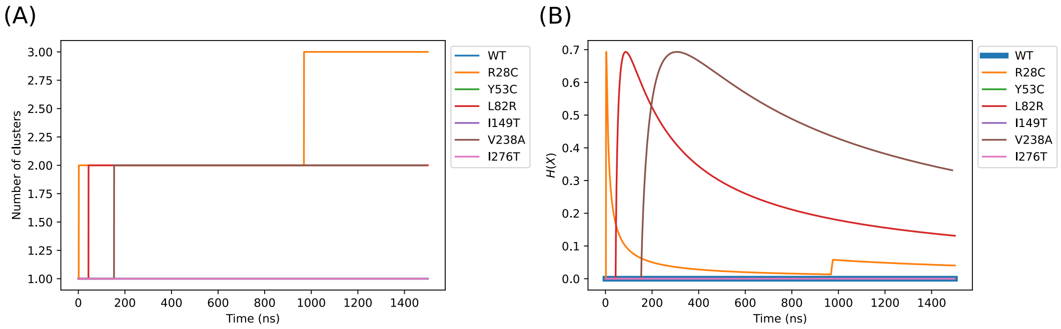

Figure S2: Analysis of the molecular dynamics simulation using the cumulated number of clusters (A) and the onstancy of the cluster entropy

the beginning of the simulation as well as an entropy value of 0. R28C, L82R, and V238A have not converged properly indicating a likely shift in the conformational space of these variants compared to the wild-type form. Since we are comparing the effect to the wild-type form, this is acceptable. However, we notice from the entropy calculation that the trajectories tend to converge at the end of the simulation.

Supplementary material 4: Fluctuation of CYP3A5 residues

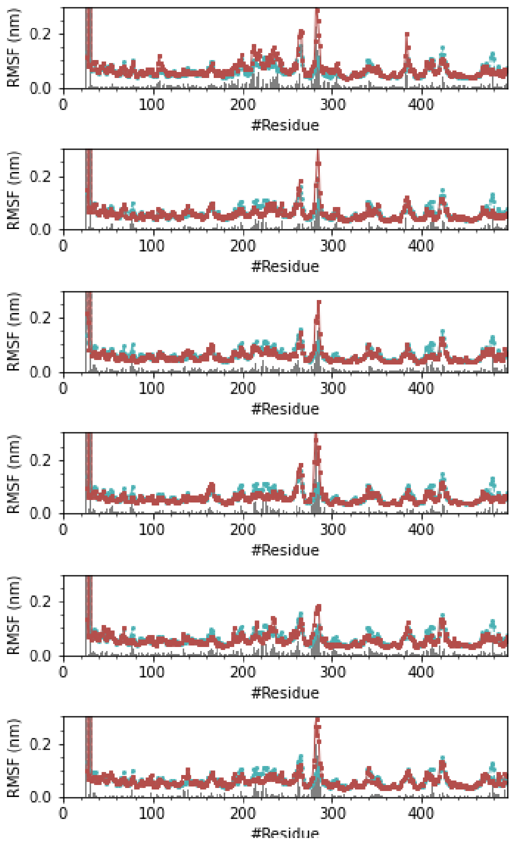

Figure S3: Root Mean Square Fluctuation of CYP3A5 residues. From top to bottom: WT, R28C, Y53C, L82R, I149T, V238A and I276T.

Supplementary material 5: Scree plot of the first 20 eigenvalues

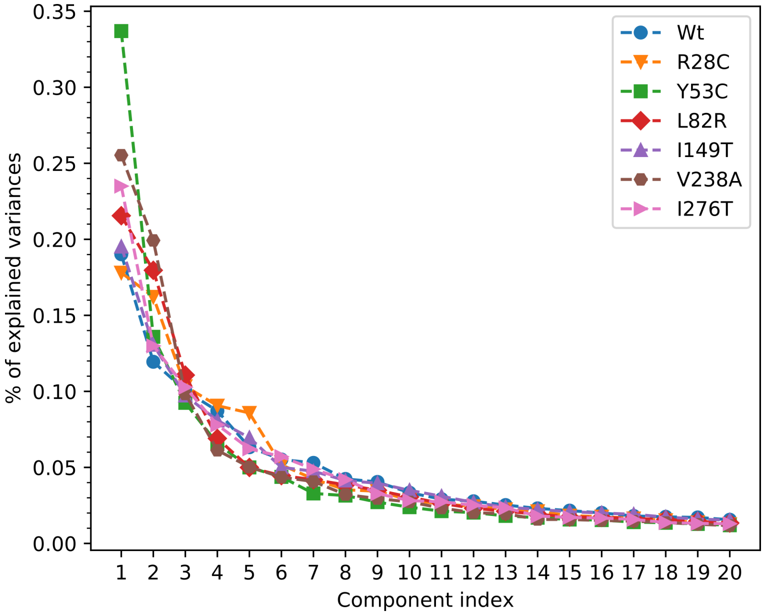

Figure S4: Scree plot showing the proportion of explained variance as a function of the component rank.

Supplementary material 6: PC3 Vs PC4

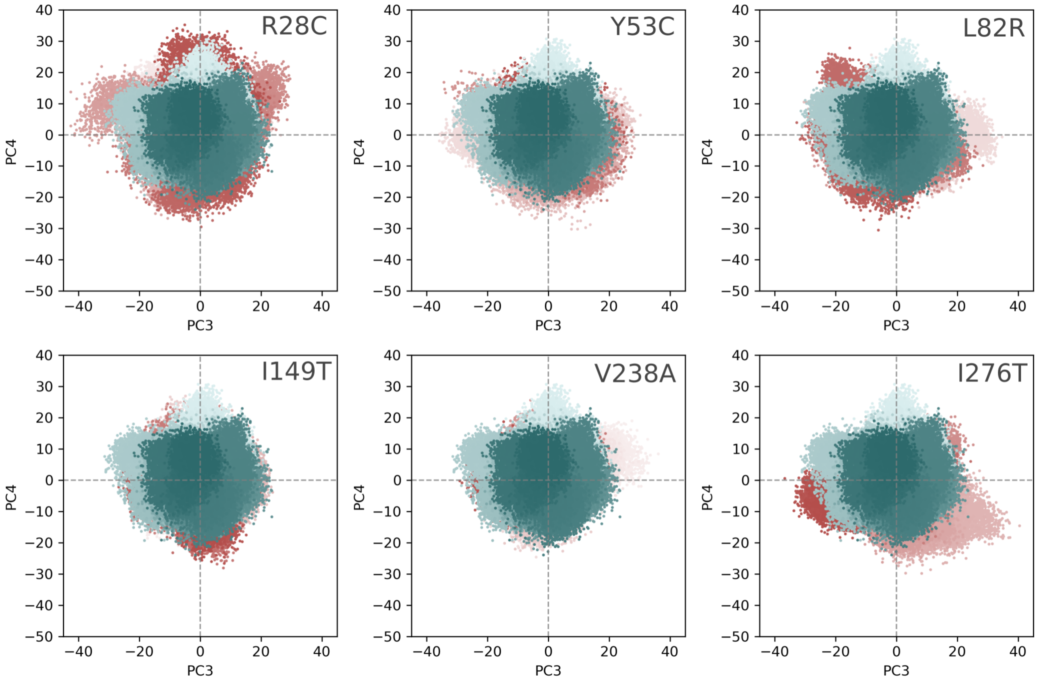

Figure S5: Principle component analysis, PC3 Vs PC4

Supplementary material 7: Porcupine Plots

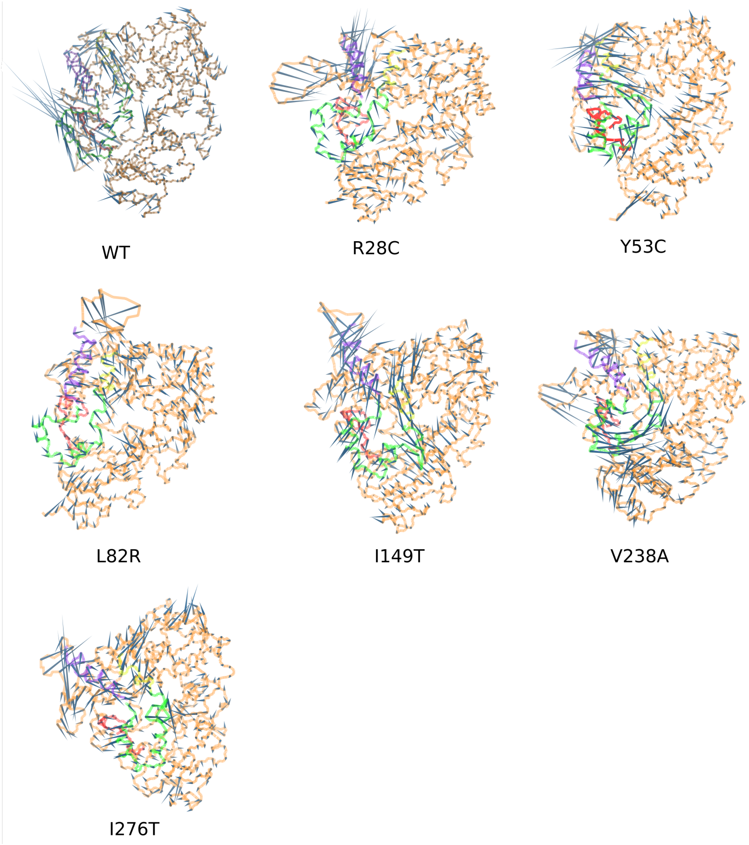

Figure S6: Porcupine plot corresponding to projections of C_α atoms onto the first three non-rotational and non-translational normal modes. The segments in green, purple, yellow and red correspond respectively to the F/G loop, F helix, G helix and the B/C loop.

Supplementary material 8: Free energy landscape of all CYP3A5 variants

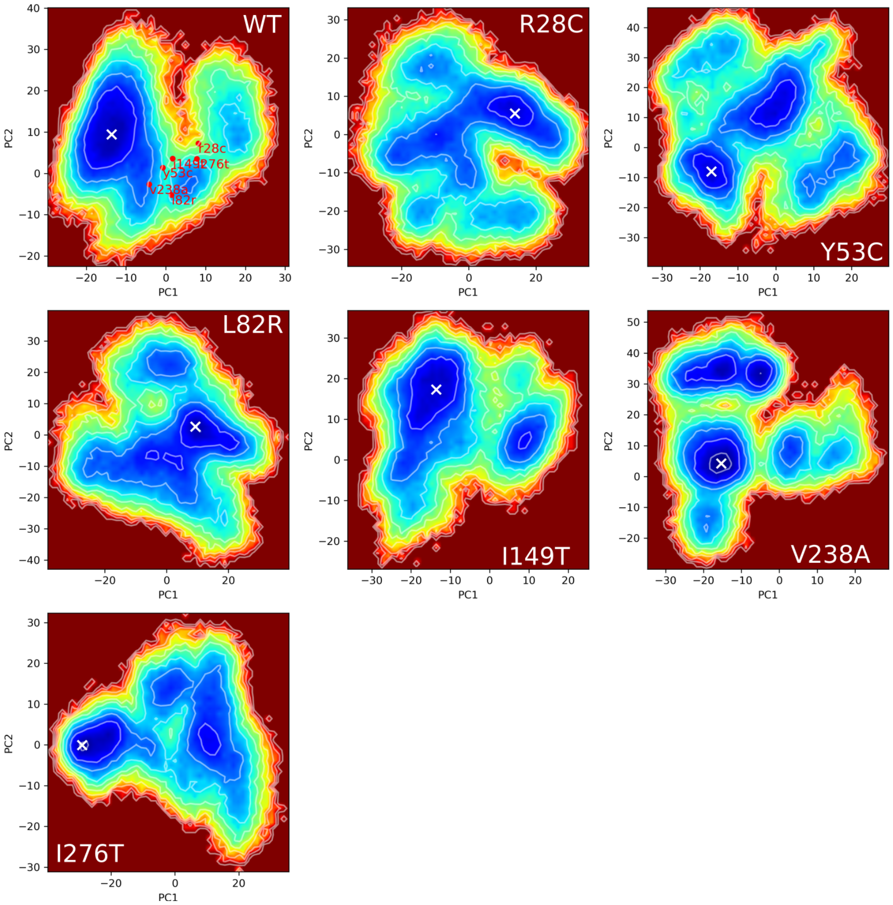

Figure S7: Free energy landscape of the wild type and CYP3A5 variants characterized from sub-Saharan African population. The white X marker indicates the position of the global minima. The position of the conformations at the local minimum of each variant was projected onto the free energy landscape of the wild-type form.

Supplementary material 9: Convergence of MM-GBSA

The convergence of the MM-GBSA calculation was verified for the complex of CYP3A5/ritonavir for the wild type form for a total simulation time of 300 ns. We plotted the MM-GBSA energy over an increasing time step of 5 ns in two fashions: forward (all the frames below the upper boundary of the intervals are considered in the calculation) and backward (all the frames above the lower boundary of the interval are considered in the calculation). We can see from the plot that the energy converges at 100 ns simulation time both for the forward and backward calculations.

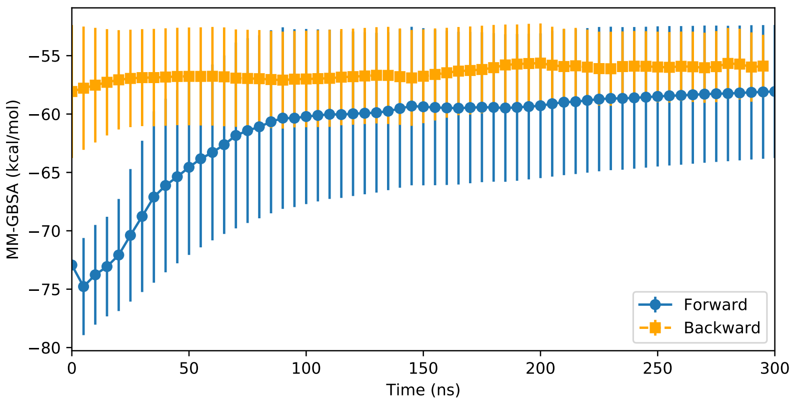

Figure S8: Convergence of the MM-GBSA energy for a simulation time of 300 ns calculated for the complex CYP3A5/ritonavir for the wild type form.
